# Supplementary material for: Development of at-home video recordings for functional skill assessment in Angelman Syndrome: a pilot study
Source: J Neurodev Disord. 2026 Feb 19;18:17. doi: 10.1186/s11689-026-09676-2 (PMC13019984; doi:10.1186/s11689-026-09676-2)
Supplement: Supplementary file 1 — Supplementary Material 1. [file 11689_2026_9676_MOESM1_ESM.docx]

| **Name** | **Eliminate** | **Keep**  **No or Minor Revisions** | **Keep Major Revisions** | **Neutral or Defer to Others** | **Comments**  (*Reminder*: Consider cultural sensitivity; meaningfulness to patients/families) |
| --- | --- | --- | --- | --- | --- |
| Reviewer |  |  |  |  |  |
| Reviewer |  |  |  |  |  |
| Reviewer |  |  |  |  |  |
| Reviewer |  |  |  |  |  |
| Reviewer |  |  |  |  |  |
| Reviewer |  |  |  |  |  |
| Reviewer |  |  |  |  |  |
| Reviewer |  |  |  |  |  |
| Reviewer |  |  |  |  |  |
| Reviewer |  |  |  |  |  |

**Key Moments for Assessment:** (Manual instructions are listed at the bottom of this form.)

- Child responds to a verbal-only question, “Do you want…?”

| **Evaluation of Task “Capture-ability”**  **(difficulty for parents, issues following instructions, difficulty getting child to cooperate, consistency of captures, etc.)** | | |
| --- | --- | --- |
| **Subj** | **How did the capture deviate from expected?** | **Notes** |
| 01 |  |  |
| 02 |  |  |
| 03 |  |  |
| 04 |  |  |
| 05 |  |  |
| 06 |  |  |
| 07 |  |  |
| 08 |  |  |
| 09 |  |  |
| 10 |  |  |
| 11 |  |  |
| 12 |  |  |

| **Evaluation of Task’s Value for Assessment**  **(types of behaviors demonstrated, behaviors that could be coded, etc.)** | | | | |
| --- | --- | --- | --- | --- |
| **What is captured, observable, scorable?** | **Key domain** | **Subjects Who Showed the Behavior** | **Subjects Who Did Not** | **Notes** |
|  | Select |  |  |  |
|  | Select |  |  |  |
|  | Select |  |  |  |
|  | Select |  |  |  |
|  | Select |  |  |  |
|  | Select |  |  |  |
|  | Select |  |  |  |
|  | Select |  |  |  |
|  | Select |  |  |  |
| Add more rows as needed. | Select |  |  |  |

| **Summary & Recommendations** |  |
| --- | --- |
| Were the key moments consistently captured? Explain. |  |
| Were *unexpected/unintended* points consistently captured? Explain. |  |
| Should the instructions be improved upon? Why? How? |  |
| Is this a good task to keep? |  |
| *Add more rows as needed.* |  |

| **Additional Thoughts/Notes:** |
| --- |
| Need to code highest communication displayed prompted and unprompted  Also need to indicate whether communication is prompted or spontaneous  Are we coding what level they understand the instruction (verbal versus object presentation)?  Consider embedding into another task. |

| **Summary from Quality:** |
| --- |
| One video rejected/reshot  Many videos do not show caregiver and there is some evidence caregivers are prompting off camera |

**Manual Instructions:**

Setup: For this video, we want to capture **how you typically determine whether your child wants something**. You can film this video with the phone in your hand, or if you need your hands free, you can have a third person film or use the tripod. For this video, you will capture an example of how your child lets you know that he/she wants something or communicates “yes” to you. Pick something your child typically enjoys, such as a favorite food (e.g., “Do you want to take a bath? Do you want a snack?”). Make sure your child has a communication device that he/she would use.

1. Start recording

2. First ask your child verbally (without gestures or without presenting the object) and wait for a response.

3. If you do not receive a response to the verbal question, follow up with whatever method you typically use to provide cues and wait for a response.

4. When you have received whatever response you expect to from your child, start doing whatever your child said “yes” to, and film your child’s reaction.

5. If your child communicates “no”, you can use this video for the “No” video and try again with a different question when you are able.
