# Supplementary material for: Development of at-home video recordings for functional skill assessment in Angelman Syndrome: a pilot study
Source: J Neurodev Disord. 2026 Feb 19;18:17. doi: 10.1186/s11689-026-09676-2 (PMC13019984; doi:10.1186/s11689-026-09676-2)
Supplement: Supplementary file 2 — Supplementary Material 2. [file 11689_2026_9676_MOESM2_ESM.pdf]

INTEGREVIEW IRB

December 31, 2020

APPROVED

Welcome

Thank you for participating in this video capture study. We really appreciate the time and effort that your family will be putting in to help us understand the range of developmental skills in individuals with Angelman syndrome (AS) that can be captured in their own homes.

The list of videos that we are asking you to take can be recorded over a two-week period. You do not need to film all the tasks at the same time. You know your child best—select times that work for your family.

If you think your child may need your help to demonstrate what he/she can do, having a third person to start and stop the recording might be helpful so that you can focus on demonstrating the task.

If you have any questions at any time, please contact your study coordinator by email at asva@casimirtrials.com or by phone at (800) 699-2704.

You can upload the videos from the camera roll of your smartphone at any time during the two-week capture period.

Videos

This manual will list the duration of each video requested, the required supplies (if any) beyond the phone and the tripod, and specific positioning of your child and the phone, if necessary.

The instructions for many of these video request that you await a response, which means that you should pause and wait for a meaningful response from your child. Try to wait until there is a clearly visible or an auditory response before you interpret what he/she is saying or before you help him/her. Make sure that your child has access to the AAC device that he/she normally uses when recording videos that involve communication and that the volume is at the maximum. Please include a view of the display at the end of the videos recorded with use of the device.

Re-Recording Videos

Only re-record a video if there is an issue during filming, such as the phone was dropped or other interruption to filming, that could get in the way of having a clear view of the activity. Before you start filming, please make sure that everything is prepared and everyone around is aware to avoid interruptions.

To make sure the study respects your time and participation, you will only be asked to re-record an activity when it is necessary to, so that the data we collect from you is consistent with the instructions in this manual.

casimir
INTEGREVIEW IRB
December 31, 2020
APPROVED

Caregiver Quick Reference

Date of Enrollment: **Date of Exit Interview:**

Final Date to Upload Videos:

	Task	Notes
☐	**Reading a Book**	
☐	**Bubble Blower**	
☐	**Ball Tower**	
☐	**Communicating "Yes"**	
☐	**Communicating "No"**	
☐	**Snack Choice**	
☐	**Greeting a Familiar Person**	
☐	**Complex Conversation**	
☐	**At the Dining Table**	
☐	**Brushing Teeth**	
☐	**Washing Hands**	
☐	**Drawing/Writing**	
☐	**Caregiver Choice Fine Motor**	

casimir
INTEGREVIEW IRB
December 31, 2020
APPROVED

☐	**Follow a 2-3 Step Routine**	
☐	**Lying in bed to getting up**	
☐	**Stand up from Floor**	
☐	**Stand Up from Couch**	
☐	**Retrieve Object from Floor**	
☐	**Walking**	
☐	**Stairs**	
☐	**Jump**	
☐	**Run**	
☐	**Caregiver Choice Gross Motor**	

INTEGREVIEW IRB
December 31, 2020
APPROVED

Reading a Book

Function

Communication skills – understanding what is said and expressing thoughts

Video Length

5 minutes

Supplies

- A familiar book or magazine that your child finds interesting

Setup

For this video, you can sit on the floor, at a table or on a couch. Set up the tripod so that you sit next to your child with both of your faces towards the camera. Please ensure that that the video captures the upper bodies and faces, including the arms, of both you and your child.

Start the recording and sit next to your child. Begin to look at the book/magazine together. If your child uses an AAC device, make sure he/she has it for this task.

Film yourself interacting with your child for around five minutes. Make sure that you include the following interactions with your child in that time:

1. Open the book and ask your child, "What is this?" (pointing to an object he/she is familiar with). Wait to see if you get a response. If you get a verbal or non-verbal response, confirm the response: "Yes, that's the [object]." Repeat this process with two additional objects.
2. Pick another object your child is familiar with in the book and ask your child, "Can you point to the [object]?" Wait to see if you get a response. If you get a response, confirm the response: "Yes, that's the [object]!" Repeat this process with two additional objects.
3. Spend the rest of the five minutes looking through the book/magazine together the way that you typically might.
4. At the end of the video, hold up the book/magazine to the camera to record what type of reading material it is.

Playing with Bubble Blowers

Function

Communication skills – understanding what is said and expressing thoughts

Video Length

5 minutes

Supplies

- Bubble blowers from the supply kit (one will be ready to function, and the other will not)

Setup

casimir
INTEGREVIEW IRB
December 31, 2020
APPROVED

For this video, you can sit on the floor, at a table or on a couch. Set up the tripod so that you sit next to your child facing each other. Please ensure that that the video captures your upper bodies, including the arms and the side of your faces.

1. Start recording.
2. Pick up the bubble blower that works and activate the bubbles Stop pulling the trigger and wait for a response to see if your child asks for more.
3. Hold up the bubble blower that does not work to your child and wait for a response to see if he/she indicates that he/she wants it.
4. Give the bubble blower that does not work to your child and wait to see if he/she can determine whether it works or not. If he/she cannot, demonstrate that the blower does not work.
5. Wait for a response.
6. Show your child again that your bubble blower works and wait for a response.
7. Spend a minute or so just playing with your bubble blower pausing to give the child time to request the blower.

Playing with the Ball Tower

Function
Communication skills – understanding what is said and expressing thoughts

Video Length
5 minutes

Supplies

- Ball tower and balls from the supply kit (one ball will not fit into the tower)

Setup
For this video, you can sit on the floor, at a table or on a couch. Set up the tripod so that you sit next to your child with both of your faces towards the camera. Please ensure that that the video captures the upper bodies and faces, including the arms, of both you and your child.

1. Start recording.
2. Show your child the balls and place them just outside of his/her reach. Wait for a response.
3. Take one of the balls that fits and put it on the tower and wait for a response.
4. Give your child that ball and let him play with it and put it down the tower.
5. Next give your child the ball that does not fit and wait for a response.
6. Demonstrate that the ball does not fit and wait for a response to see if your child indicates that he/she wants the other balls that fit.
7. Engage in back and forth play with the ball tower and correct ball or an additional minute or so.

Communicating Yes

Function

INTEGREVIEW IRB
December 31, 2020
APPROVED

Communication skills – understanding what is said and expressing thoughts

Video Length
Up to 2 minutes

Setup
For this video, we want to capture how you typically determine whether your child wants something. You can film this video with the phone in your hand, or if you need your hands free, you can have a third person film or use the tripod. For this video, you will capture an example of how your child lets you know that he/she wants something or communicates "yes" to you. Pick something your child typically enjoys, such as a favorite food (e.g., "Do you want to take a bath? Do you want a snack?"). Make sure your child has a communication device that he/she would use.

1. Start recording
2. First ask your child verbally (without gestures) and wait for a response.
3. If you do not receive a response to the verbal question, follow up with whatever method you typically use to provide cues and wait for a response.
4. When you have received whatever response you expect to from your child, start doing whatever your child said "yes" to, and film your child's reaction.
5. If your child communicates "no", you can use this video for the "No" video and try again with a different question when you are able.

Communicating No

Function
Communication skills – understanding what is said and expressing thoughts

Video Length
Up to 2 minutes

Setup
For this video, we want to capture how you typically determine whether your child does not want something. You can film this video with the phone in your hand, or if you need your hands free, you can have a third person film or use the tripod.

For this video, you will capture an example of how your child lets you know that he/she does not want something or communicates "no" to you.

Please think of a question you might ask your child in your daily life about his/her preferences that you think he/she will indicate "no" to, such as food or TV show he/she doesn't like.

1. Start recording.
2. First ask your child verbally (without gestures) and wait for a response.
3. If you do not receive a response to the verbal question, follow up with whatever method you typically use to provide cues and wait for a response.
4. When you have received whatever response you expect to from your child, take away or turn off whatever your child said "no" to, and film your child's reaction.

INTEGREVIEW IRB
December 31, 2020
APPROVED

5. If your child communicates “yes”, you can use this video for the previous task and try again with a different question when you are able.

Choosing Between Two Options

Function

Communication skills – understanding what is said and expressing thoughts

Video Length

Up to 2 minutes

Setup

For this video, we want to capture how you typically determine your child’s preference between two choices. You can film this video with the phone in your hand, or if you need your hands free, you can have a third person film or use the tripod.

Please think of a choice you might typically ask your child to make, such as between two snack options, two toys, two books, or anything else that might come up in daily life.

1. Start recording.
2. First ask your child to choose verbally (without gestures or visual cues, like holding up the objects) and wait for a response.
3. If you do not receive a response to the verbal question, follow up with whatever method you typically use to provide cues and wait for a response.
4. When you have received whatever response you expect to from your child, provide your child with his/her choice, and film your child’s reaction.

Greeting a Familiar Person

Function

- Spontaneous communication
- Interaction with loved ones

Video Length

Up to 5 minutes

For this video, capture an example of how your child greets and interacts with someone he/she is familiar with and would be excited to see, such as a family member returning home or coming to visit. Film your child as this person comes in the room, greets, and then interacts with your child.

Complex Conversation/Interaction

Function

INTEGREVIEW IRB
December 31, 2020
APPROVED

Communication skills – understanding what is said and expressing thoughts

For these videos, capture some situations where your child is the most communicative and demonstrates his/her highest communication skills. These exchanges might involve verbal, gestures, eye gaze, signing, or a combination of multiple ways of communicating. The videos in this section are intended to capture what you feel represents your child's best abilities. They can also be submitted in different segments; it does not have to be all one long video.

At the Table

Function

Fine motor skills

Video Length

Up to 5 minutes

Supplies

- Spoon and food that is typically eaten with a spoon
- Fork and food that can be stabbed with a fork
- A cup he/she regularly drinks from filled with a beverage he/she enjoys
- Small handful of finger foods

Setup

Have your child seated in the location he/she typically eats a meal. Either film the child with the phone in your hand as you sit facing your child or sit facing your child with the phone on the tripod right behind you. Make sure that the phone can record your child's upper body, including his/her arms, in the shot.

1. Start recording.
2. Place the food that requires a spoon in front of your child.
3. Give your child time to eat whatever he/she can independently.
4. If your child does not pick up the spoon, try placing the spoon in his/her hand and wait to see if he/she can use it.
5. If your child is unable to use the spoon, assist him/her to eat however you typically do during meals.
6. After a few bites, remove the "spoon" food and replace it with the "fork" food.
7. Give your child time to eat whatever he/she can independently.
8. If your child does not pick up the fork, try placing the fork in his/her hand and wait to see if he/she can use it.
9. If your child is unable to use the fork, assist him/her to eat however you typically do during meals.
10. Place the cup in front of your child.
11. Give your child time to drink independently.
12. If your child is unable to drink, assist him/her to drink however you typically do during meals.
13. Place a few of the finger food items on the table in front of your child. Allow your child to eat the finger-foods as he/she is able.
14. When your child has eaten all the placed items, ask him/her, "Do you want some more?" Wait for a response.

INTEGREVIEW IRB
December 31, 2020
APPROVED

15. If you do not get a response, use whatever cues you can to ask if he/she wants more. Wait for a response.
16. Put more of the food items on the table regardless of whether you get a response.
17. Give your child time to see if he/she shares with you.
18. If he/she does not share, ask your child, "Can I have a [Cheerio/marshmallows]?" Wait for a response.
19. If you do not get a response, use whatever cues you can (gestures, etc.) to ask if he/she will share. Wait for a response.

Brushing Teeth

Function

- Fine motor skills
- Self-care skills

Video Length

Up to 5 minutes

Supplies

- Toothbrush and toothpaste

Setup

Place the phone in a location that allows a view of your child's face and upper body. If this task is done at a sink with a mirror, you can video into the mirror at the reflected view of your child. If you cannot set the phone up on the tripod because of space constraints, you may need a third person to record while you help your child.

For this task, you will video your child brushing his/her teeth while seated in his/her wheelchair/chair or standing—however and wherever he/she normally does this task.

Teeth brushing

1. Start recording.
2. Put toothpaste on your child's toothbrush for them.
3. Ask your child to brush his/her teeth (with words, gestures, demonstrating – any strategy except physically assisting him/her) and give him/her time to do as much as he/she can without help.
4. If you typically help your child brush, assist him/her however you typically do.

Washing Hands

Function

- Fine motor skills
- Self-care skills

Video Length

Up to 5 minutes

Supplies

- Soap

casimir
INTEGREVIEW IRB
December 31, 2020
APPROVED

Setup
Place the phone in a location that allows a view of your child's face and upper body. If this task is done at a sink with a mirror, you can video into the mirror at the reflected view of your child. If you cannot set the phone up on the tripod because of space constraints, you may need a third person to record while you help your child.

For this tasks, you will video your child washing his/her hands while seated in his/her wheelchair/chair or standing—however and wherever he/she normally does this task.

Hand washing
1. Start recording.
2. Ask your child to wash his/her hands (with words, gestures, demonstrating – any strategy except physically assisting him/her) and give him/her time to do as much as he/she can without help.
3. If you typically help your child wash, assist him/her however you typically do.

Drawing/Writing

Function
Fine motor skills

Video Length
Up to 5 minutes

Supplies
- Blank paper
- Pen or crayon – whichever you think would be easier for your child to use and hold

Setup
For this video, you can sit on the floor, at a table or on a couch. Set up the tripod so that you sit next to your child with both of your faces towards the camera. Please ensure that that the video captures the upper bodies and faces, including the arms, of both you and your child. For each object you ask your child to draw, you can demonstrate what you're asking for with your own pen. Wait to see if your child is able to complete the task independently before offering any assistance, such as hand-over-hand.

1. Start recording.
2. Ask or demonstrate that your child draws a line.
3. Ask or demonstrate that your child draws a circle.
4. Ask or demonstrate that your child draws a square.
5. Ask or demonstrate that your child draws a letter other than O.
6. Ask or demonstrate that your child draws a person.

Caregiver Choice – Fine Motor

Function
Fine motor skills

INTEGREVIEW IRB
December 31, 2020
APPROVED

Video Length
Up to 2 minutes

For this task, think about a fine motor activity that your child struggles with or might need assistance with. The idea for this video is that if your child received a treatment that improved his/her hand and arm function, this series would demonstrate what that activity was like before and after treatment. That means it's not a bad thing to record something your child is unable to do but might be able to with a small amount of improvement in function.

Following a Routine

Function

- Communication skills
- Fine motor skills
- Self-care skills

Video Length
Up to 5 minutes

For the next video, you will film the process of getting your child ready to leave the home. Please record this video when you are leaving for an outing that your child is excited to go on. If your child needs assistance, have a third person film or use the tripod. Film all steps in the routine, such as putting your shoes on, getting your coat, coming to the door.

Lying in Bed to Getting Up

Function

- Gross motor skills
- Self-care skills

Video Length
Up to 2 minutes

For the following activity, you will video your child going from lying down in bed to sitting up on the side of the bed or standing up. You can video this task as part of your regular morning routine, or set-up at another time of day. Make sure all the blankets and sheets are removed from your child before he/she sits up. Also make sure that you can see your child's entire body in the frame.

1. Start recording.
2. While your child is lying on his/her back in bed, say something like, "Let's get up!" Give your child time to respond.
3. If your child is unable or does not seem to understand your request, use whatever cues that might help, such as reaching your hand out, pulling on his/her hands. Let your child perform as much of the task by themselves as able.

INTEGREVIEW IRB
December 31, 2020
APPROVED

4. If your child needs physical assistance to get out of bed, help him/her as you typically would.

Stand up from floor

Function

Gross motor skills

Video Length

Up to 1 minute

Setup

For this video, make sure that you can see your child's entire body in the frame when both sitting and standing.

1. Start recording.
2. Have your child sit on the floor either independently or alongside you if you need to demonstrate what to do.
3. Prompt your child to stand up (with words, gestures, demonstrating – any strategy except physically assisting him/her).
4. If your child needs physical assistance to get up, help him/her as you typically would.

Stand up from couch

Function

Gross motor skills

Video Length

Up to 1 minute

Setup

For this video, make sure that you can see your child's entire body in the frame when both sitting and standing. Have your child sit in the middle of the couch cushion.

1. Start recording.
2. Have your child sit on the couch either independently or alongside you if you need to demonstrate what to do.
3. Prompt your child to stand up (with words, gestures, demonstrating – any strategy except physically assisting him/her).
4. If your child needs physical assistance to get up, help him/her as you typically would.

Retrieve object from floor

Function

Gross motor skills

casimir
INTEGREVIEW IRB
December 31, 2020
APPROVED

Video Length
Up to 1 minute

Setup
For this video, make sure that you can see your child's entire body in the frame when bending over and standing up.

1. Start recording.
2. Place one of the balls from the ball tower toy on the floor in front of your child.
3. Prompt your child to pick up the ball (with words, gestures, demonstrating – any strategy except physically assisting him/her).
4. If your child is not physically able or does not seem to understand your request, help him/her as you typically would.

Walking

Function
Gross motor skills

Video Length
Up to 2 minutes

For this task, you will take two sets of videos: one of your child walking around inside the home and one of your child walking around outside. For the outside video, make sure that other people are not in the video and the ground is level. Ideally, you would capture them from the front for about 10 steps while he/she is walking toward you and from the side for about 10 steps. If your child typically uses braces (e.g. afos/smos) or a walking aid (e.g. a walker) in either location, he/she should use those in the video. To maintain steadiness in the recording, it is best to try and capture your child while you are standing still and just pan the camera to follow your child, if possible. Also make sure that you can see your child's entire body in the frame.

Stairs

Function
Gross motor skills

Video Length
Up to 2 minutes

For this task, you will first record your child walking at least 5 steps up a flight of stairs. Ideally, you would capture the video from the top of the stairs as your child walks toward you. You will then record your child walking at least 5 steps down the flight of stairs, ideally recorded from the bottom of the stairs as your child walks toward you. If there are concerns with safety, please have someone standing close to your child as he/she walks up the stairs. He/she can use the handrail or wall as needed for support. If the only way your child can go up the stairs safely is by holding your hands, you can provide that assistance. Only have your child

INTEGREVIEW IRB
December 31, 2020
APPROVED

perform this task if you feel it is safe for them to do so. Also make sure that you can see your child's entire body in the frame.

Jump

Function

Gross motor skills

Video Length

Up to 2 minutes

For this task, you will record your child jumping. Make sure that you can see your child's entire body in the frame.

1. Start recording.
2. Prompt your child to jump up and down (with words, gestures, demonstrating – any strategy besides physically assisting him/her).
5. If your child is not able or does not to understand your request, help him/her as you typically would to see if he/she can jump with assistance.

Run

Function

Gross motor skills

Video Length

Up to 2 minutes

For this task, you will take two sets of videos: one of your child running inside the home and one of your child running around outside. For the outside video, make sure that other people are not in the video and that the ground is level. Ideally, you would capture them from the front for about 10 steps while he/she is running toward you and from the side for about 10 steps. If your child typically uses braces or a walking aid (e.g. a walker) in either location, he/she should use those in the video. To avoid the video shaking, it is best to try and capture your child while you are standing still and just panning the camera to follow your child, if possible. Also make sure that you can see your child's entire body in the frame.

One strategy to try to see if your child is able to run, try inviting him/her to a favorite activity, such as playing with water. "Let's go play with the water table!" Record whether your child picks up speed over a walk.

Caregiver Choice – Gross Motor

Function

Gross motor skills

casimir
INTEGREVIEW IRB
December 31, 2020
APPROVED

Video Length
Up to 2 minutes

For this task, think about a gross motor activity that your child struggles with or might need assistance with. The idea for this video is that if your child received a treatment that improved his/her gross motor function, this series would demonstrate what that activity was like before and after treatment. That means that it's not a bad thing to record something your child is unable to do but might be able to with a small amount of improvement in function.

INTEGREVIEW IRB

December 31, 2020

APPROVED

Using ShareFile

Registering for the App

For this study, you'll be using the ShareFile mobile app to capture the video assessments. Casimir study staff will set up a time to help get you registered for the app. Look for the registration email from ShareFile Support (contact Casimir at 1-800-881-5657 if you have not received the email) and follow the steps below:

Step 1

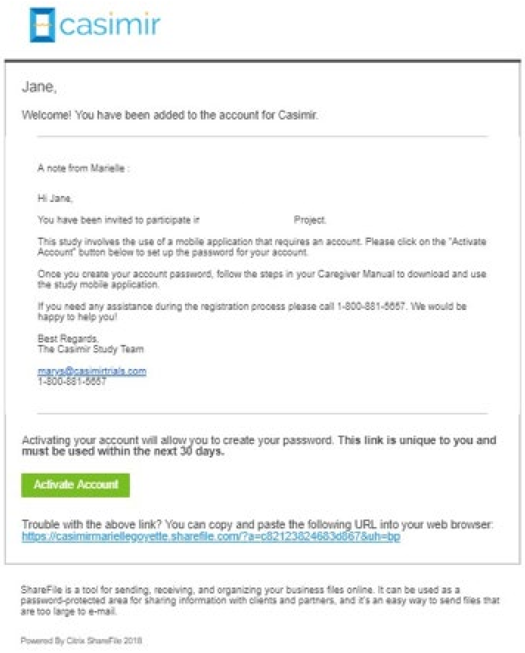
casimir

Jane,

Welcome! You have been added to the account for Casimir.

A note from Marielle :

Hi Jane,

You have been invited to participate in Project.

This study involves the use of a mobile application that requires an account. Please click on the "Activate Account" button below to set up the password for your account.

Once you create your account password, follow the steps in your Caregiver Manual to download and use the study mobile application.

If you need any assistance during the registration process please call 1-800-881-5657. We would be happy to help you!

Best Regards,
The Casimir Study Team

marys@casimirtrials.com
1-800-881-5657

Activating your account will allow you to create your password. **This link is unique to you and must be used within the next 30 days.**

Activate Account

Trouble with the above link? You can copy and paste the following URL into your web browser:
https://casimirmariellegoyette.sharefile.com/?a=c82123824683d867&uh=bp

ShareFile is a tool for sending, receiving, and organizing your business files online. It can be used as a password-protected area for sharing information with clients and partners, and it's an easy way to send files that are too large to e-mail.

Powered By Citrix ShareFile 2018

Click on "Activate Account".

Step 2

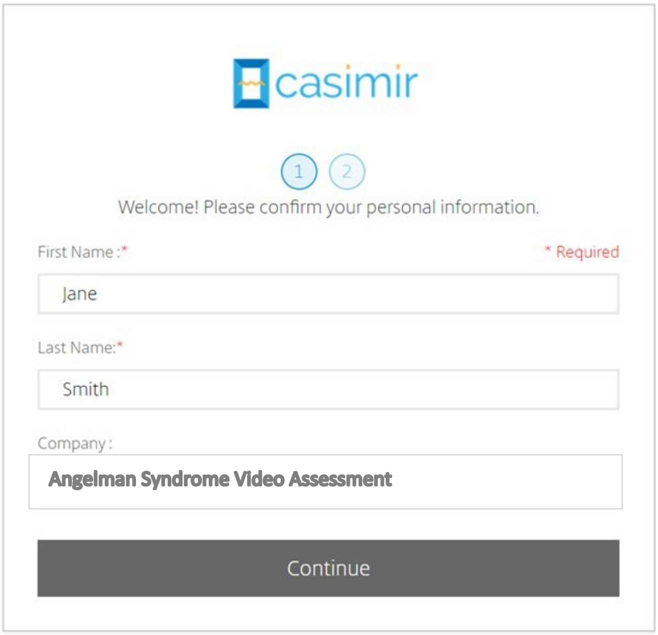

Check the information for accuracy and click "Continue".

INTEGREVIEW IRB

December 31, 2020

APPROVED

Step 3

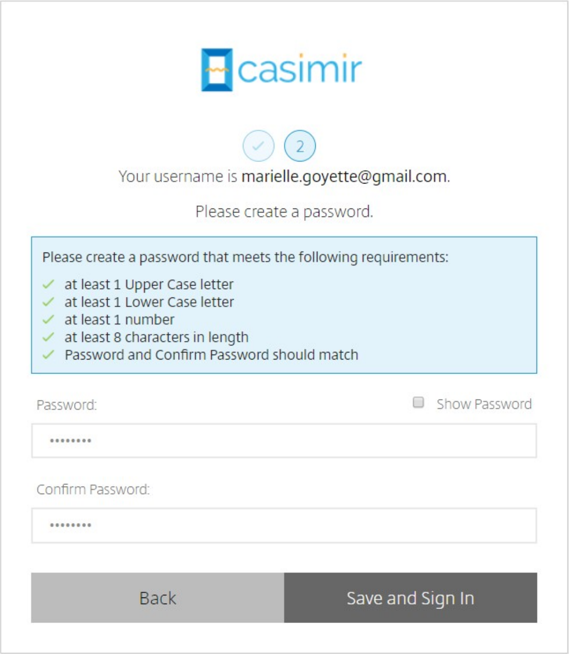

Chose a password, confirm the password, and click “Save and Sign in”.

Step 4

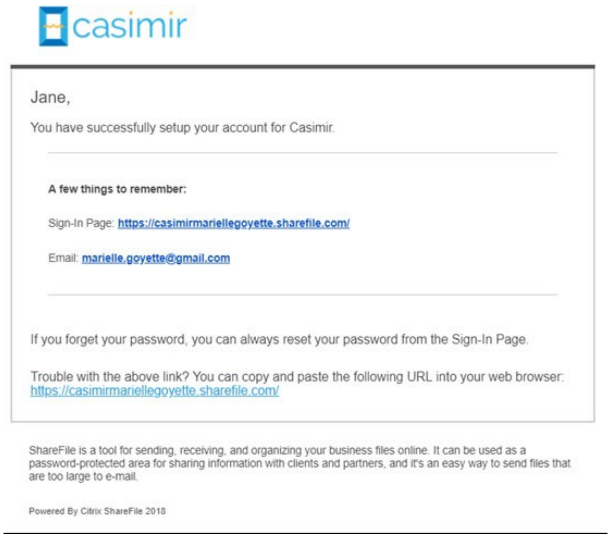

Registration complete.

INTEGREVIEW IRB

December 31, 2020

APPROVED

Downloading the App

Step 1

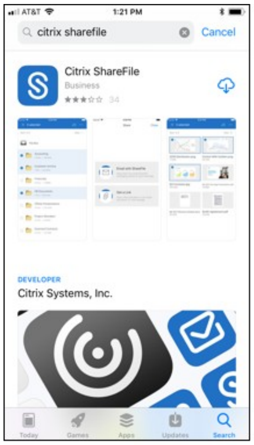

Download "Citrix ShareFile" from the Apple App Store or Google Play Store

Step 2

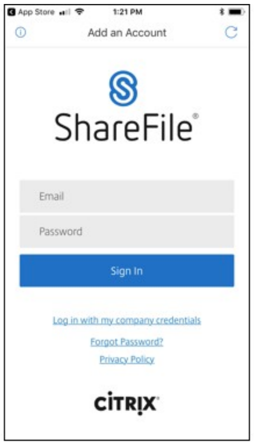

Enter your email address and the password you created during registration

Step 3

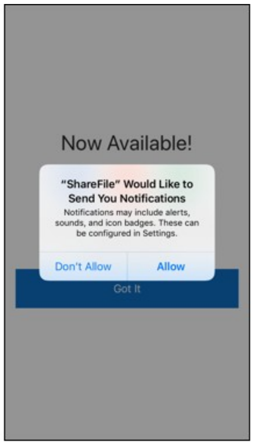

Allow ShareFile to send you notifications

Step 4

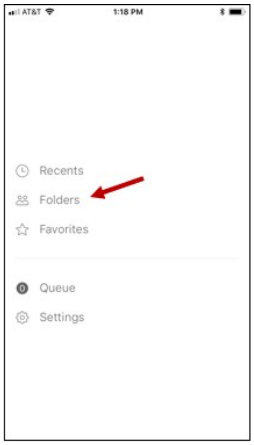

Go to Folders

Step 5

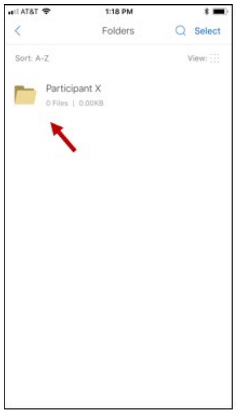

Click on your participant folder

Step 6

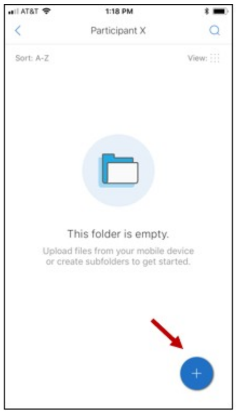

Click on the blue plus button in the bottom righthand corner

Step 7

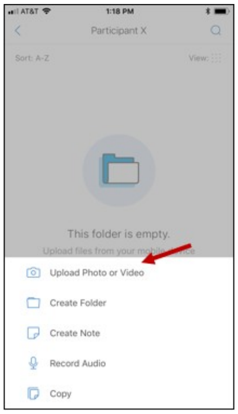

Select Upload Photo or Video

Step 8

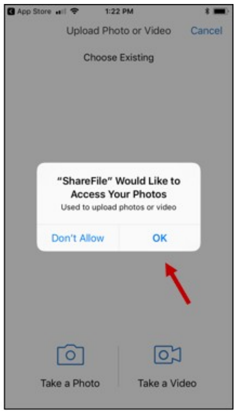

Allow ShareFile to Access your Photos

INTEGREVIEW IRB

December 31, 2020

APPROVED

Upload a Video to ShareFile

Step 1

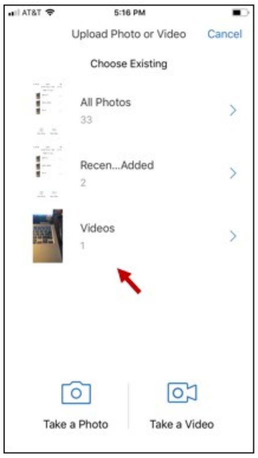

Click on Videos.

Step 2

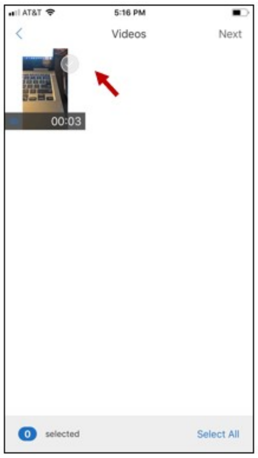

Click on the video you would like to upload.

Step 3

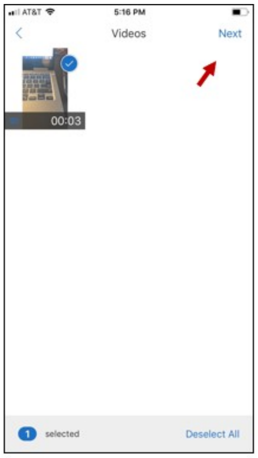

Click Next.

Step 4

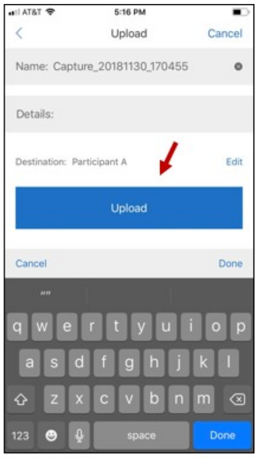

Keep the name labeled with the date and time of video capture. Click Upload.

Step 5

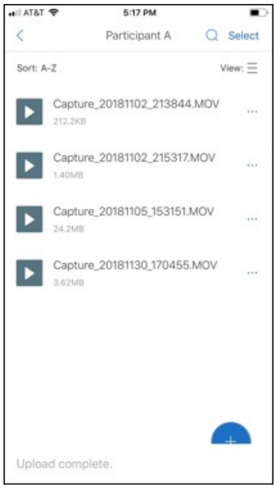

Your upload is complete!
